# Whole genome sequencing of low input circulating cell‐free DNA obtained from normal human subjects

**DOI:** 10.14814/phy2.14993

**Published:** 2021-08-04

**Authors:** Julie F. Foley, Brian Elgart, B. Alex Merrick, Dhiral P. Phadke, Molly E. Cook, Jason A. Malphurs, Gregory G. Solomon, Ruchir R. Shah, Michael B. Fessler, Frederick W. Miller, Kevin E. Gerrish

**Affiliations:** ^1^ Division of National Toxicology Program NIEHS Durham North Carolina USA; ^2^ Division of Intramural Research NIEHS Durham North Carolina USA; ^3^ Sciome LLC Durham North Carolina USA

**Keywords:** circulating cell‐free DNA, genomic sequencing, plasma DNA, variants

## Abstract

Cell‐free DNA circulates in plasma at low levels as a normal by‐product of cellular apoptosis. Multiple clinical pathologies, as well as environmental stressors can lead to increased circulating cell‐free DNA (ccfDNA) levels. Plasma DNA studies frequently employ targeted amplicon deep sequencing platforms due to limited concentrations (ng/ml) of ccfDNA in the blood. Here, we report whole genome sequencing (WGS) and read distribution across chromosomes of ccfDNA extracted from two human plasma samples from normal, healthy subjects, representative of limited clinical samples at <1 ml. Amplification was sufficiently robust with ~90% of the reference genome (GRCh38.p2) exhibiting 10X coverage. Chromosome read coverage was uniform and directly proportional to the number of reads for each chromosome across both samples. Almost 99% of the identified genomic sequence variants were known annotated dbSNP variants in the hg38 reference genome. A high prevalence of C>T and T>C mutations was present along with a strong concordance of variants shared between the germline genome databases; gnomAD (81.1%) and the 1000 Genome Project (93.6%). This study demonstrates isolation and amplification procedures from low input ccfDNA samples that can detect sequence variants across the whole genome from amplified human plasma ccfDNA that can translate to multiple clinical research disciplines.

## Graphical abstract

Plasma DNA studies frequently employ targeted amplicon deep sequencing platforms due to limited concentrations (ng/ml) of ccfDNA in the blood. This study demonstrates isolation and amplification procedures from low input ccfDNA samples that can detect sequence variants across the whole genome from amplified human plasma ccfDNA that can translate to multiple clinical research disciplines.

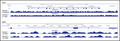

## 1 INTRODUCTION

Short extracellular DNA fragments (~170 bp) normally circulate in blood at low concentrations (ng/ml). Programmed cell death in hematopoietic cells and tissues (normal turnover) is the primary source of extracellular nuclear and mitochondrial DNA (mtDNA) in whole blood. Circulating cell‐free DNA was first described in the late 1940's (Mandel & Metais, 1948). However, in the past decade, a growing number of investigators have recognized quantitation and sequence analysis of ccfDNA as an emerging, blood‐based biomarker for disease diagnosis, staging, and therapeutic decisions in oncology (Crowley et al., 2013; Otandault et al., 2019; Suraj et al., 2016), fetal aneuploidy disorders (Breveglieri et al., 2019; Zhang et al., 2019), organ transplantation (Hayward & Chitty, 2018; Oellerich et al., 2015), autoimmune disorders (Duvvuri & Lood, 2019; Tug et al., 2014), and other pathologies (Cerne & Bajalo, 2014; Haghiac et al., 2012; Hamaguchi et al., 2015). Since ccfDNA is limited in quantity and heterogeneous in size (multiples of ~170 bp), concentrations can be highly variable, and even with the use of successful downstream methods, detecting sequence variants at a whole genome level can be challenging.

Targeted amplicon sequencing has been the preferred DNA sequencing application because when compared to tissue genomic DNA, extracted ccfDNA concentrations are low and not sufficient for WGS (Plagnol et al., 2018; Zakrzewski et al., 2019; Zhang et al., 2019). However, a targeted approach precludes gene discovery and constrains variant detection to a select few candidate genes for tissue‐specific diseases, primarily cancer. Here, we report procedures for isolation, amplification, and sequencing of plasma ccfDNA from two normal human plasma donors followed by the analysis of ccfDNA yield and quality performance metrics. These procedures should not be limited to WGS and allow for exome sequencing and candidate gene enrichment. Population databases such as COSMIC, Catalogs of Mutations in Cancer (COSMIC, Catalogue of Somatic Mutations in Cancer http://cancer.sanger.ac.uk/cosmic, Accessed 22 Aug 2017) enable comparisons of ccfDNA sequencing data against curated mutations for disease‐specific molecular signatures. Here, we describe procedures for the extraction of ccfDNA from human plasma, sequencing of the whole genome, and analysis for genomic alterations. Development of these isolation and amplification procedures from low input ccfDNA is critical when clinical plasma volume samples are limiting to 1 ml or less.

## 2 MATERIALS AND METHODS

### 2.1 Sample collection

Circulating cfDNA was extracted from healthy, de‐identified volunteers recruited from the NIEHS Clinical Research Unit (Research Triangle Park). The Institutional Review Board of NIEHS approved the protocol for this study and methodologies conformed to the standards set by the Declaration of Helsinki.

Peripheral whole blood was collected from two healthy male volunteers in Streck Cell‐Free DNA BCT® tubes (La Vista), and the plasma was separated within 2 h of collection by a double centrifugation protocol. The first spin at 1600× g was performed at room temperature for 10 min in a mega‐centrifuge (Sorvall RT7, Thermo Fisher Scientific). Following careful plasma separation, centrifugation was repeated at 16,000× g for 10 min in a benchtop micro‐centrifuge (Eppendorf 5415D, Hauppauge), and the clean plasma was transferred to a cryovial for storage at −80°C until ccfDNA isolation.

### 2.2 DNA extraction, quantification, and fragment analyses

Circulating cfDNA was extracted from 1 ml of plasma using a magnetic bead‐based kit (Maxwell® RSC ccfDNA Plasma Kit, Promega) and eluted with nuclease‐free water (50 μl volume) on a Promega Maxwell® RSC instrument. Sample yield was determined by a fluorometric method (Quantus™ Fluorometer, Promega). Fragmentation pattern analysis (FA) was used to assess the plasma DNA quality to verify the presence of characteristic ccfDNA short bp fragments of ~170 bp and the absence of genomic contamination (5200 Fragment Analyzer System, Agilent Inc.). Purified samples were stored at −80°C.

### 2.3 Library preparation and sequencing

Amplification was required to generate sufficient amounts of DNA for WGS. The initial step of library preparation typically begins with DNA shearing. Since ccfDNA is comprised of short base pair fragments (<200), it was not necessary to perform this step. Amplified sequencing libraries were prepared using a three‐step library preparation protocol for sequencing plasma ccfDNA (Table 1). Briefly, 10 μl of eluted ccfDNA at 0.1 ng/μl was used for template preparation and library synthesis. Amplification was performed in a single tube with the SMARTer ThruPLEX® Plasma Seq Library Preparation protocol according to the manufacturer's instructions (Takara Bio USA). NGS libraries were purified with Agencourt AMPure XP beads (Beckman Coulter Genomics), then amplified by a round of PCR (14 cycles) and tagged with Illumina‐compatible indexes for multiplex sequencing (SMARTer® DNA HT Dual Index Kit, Takara Bio USA). Fragment analysis of the libraries for each sample showed a median sample size of 310 bp (170 bp ccfDNA plus 140 bp adapters) containing library products of the mononucleosomal DNA fragments. Libraries were sequenced on a NovaSeq™ 6000 (Illumina) S1 platform by the NIEHS Epigenomics Sequencing Core (Research Triangle Park) at an average of 20X coverage from 2 × 150 bp paired‐end reads. Sequences were output in FASTQ format. Sequencing reads for all samples have been deposited in NCBI SRA (SUB9071852).

**TABLE 1:** Plasma ccfDNA sample summary

Sample	Pre‐amplification concentration (ng/ul)	ccfDNA input	PCR cycles	Post‐amplification clean up concentration (ng/uL)	Library size	Fold enrichment
1	0.1	1 ng	14	13	340 bp	650
2	0.1	1 ng	14	11	340 bp	550

### 2.4 Bioinformatics analysis

Sequence data quality was evaluated by the FASTQC tool v0.11.8 (www.bioinformatics.babraham.ac.uk/projects/fastqc/). Genome Analysis Toolkit (GATK) workflow describes the data pre‐processing procedures and identification of germline short variants (single nucleotide polymorphisms [SNPs] and indels). Read pairs were mapped to the human reference genome (GRCh38.p2) using the BWA alignment tool (v0.7.12) (Li & Durbin, 2009). Duplicate reads were removed using the MarkDuplicates program from Picard tools v1.99 (http://broadinstitute/github/io/picard). Aligned read numbers were obtained after duplicate reads and reads aligning on two different chromosomes were removed. Utilities from the BEDTools package and custom summarization scripts were used to obtain the coverage at each hg38 genome base (McKenna et al., 2010). Genomic coverage was summarized at the following different levels: 1X, 10X, 20X, 30X, 50X, and 100X. To identify the presence of bias during the sequencing workflow, percent aligned reads were normalized by chromosome length. In addition, using an in silico approach, coverage was also evaluated specifically at all coding regions as annotated for a commercial human whole exome sequencing library (Agilent SureSelect® Human All Exon V7 probe design). Exon probes targeted approximately 214,000 coding exons included in RefSeq (99.3%), GENCODE v24 (99.6%), CCDS (99.6%), and UCSC databases for known genes (99.6%).

Tools from GATK version 4.1.2 were used for data pre‐processing, variant calling, and filtering of variants. Base quality scores were recalibrated using the GATK tools, BaseRecalibrator, and ApplyBQSR. Identification of germline variants was performed using GATK's HaplotypeCaller (Poplin et al., 2017). Raw variants were filtered using GATK tools VariantRecalibrator and ApplyVQSR (Depristo et al., 2011; Van der et al. 2013). Variants in hg38 repeat regions or blacklisted regions were removed. Functional annotation of the variants was carried out using Snpeff version 4.3t (Cingolani et al., 2012).

Additional analyses were conducted to further characterize the sample variants. We excluded the low impact variants and then generated a mutation frequency spectrum. The analysis was performed with the R package, SomaticSignatures (v3.6; Bioconductor) (Gehring et al., 2015). Finally, we compared the identified variants with two human germline variant databases aligned against the reference genome GRCh38.p2: genome Aggregation Database, gnomAD, (v3.1.1, Broad), (Karczewski et al., 2020), and The International Genome Sample Resource, 1000 Genomes Project (Clarke et al., 2017).

## 3 RESULTS

The human genome was sequenced after the amplification of ccfDNA (1ng) using a sequencing library preparation procedure specifically designed for circulating short ccfDNA bp fragments extracted from plasma. Amplified fragments were sequenced on an Illumina NovaSeq™ 6000, S1 Sequencing System (San Diego, CA) from two unique human blood‐based samples.

### 3.1 ccfDNA yield

Amplification was performed in a single tube to reduce the possibility of contamination and loss of ccfDNA. Following the manufacturer's recommendation (Takara Bio) of 14 cycles for low input ccfDNA concentrations (ng/μl), we observed a ccfDNA enrichment of greater than 500‐fold (Table 1). Post‐amplification fragment analysis demonstrated 340 bp library size without contamination for WGS.

### 3.2 Performance metrics

Paired‐end sequencing was performed and aligned across the hg38 genome build. The mean number of sequencing reads was 577.7 million. Aligned mapped reads for samples 1 and 2 were 53% and 73% to the GRCh38.p2 reference genome (Table 2). Decreased alignment observed in sample 1 was attributed to artifact of unknown origin that compromised the sample. A PCR bubble present in the FA of sample 1 indicated slight over amplification that likely accounts for the increased read duplicate numbers for that sample. A reasonable duplication rate (25%) was observed in the other sample. After removal of the duplicate reads, and reads aligning on two different chromosomes, a reliable number of reads was achieved for alignment to the reference genome for both samples.

**TABLE 2:** Summary performance statistics for WGS from amplified ccfDNA

Sample ID	Read length	Total reads (Million)	Aligned reads	Alignment rate	Duplicate reads
1	PE−150^a^	857.9	456.7	53%	45%
2	PE−150^a^	955.4	699.3	73%	25%

^a^ PE‐150: Paired‐end, 150 base pairs

**FIGURE 1:**
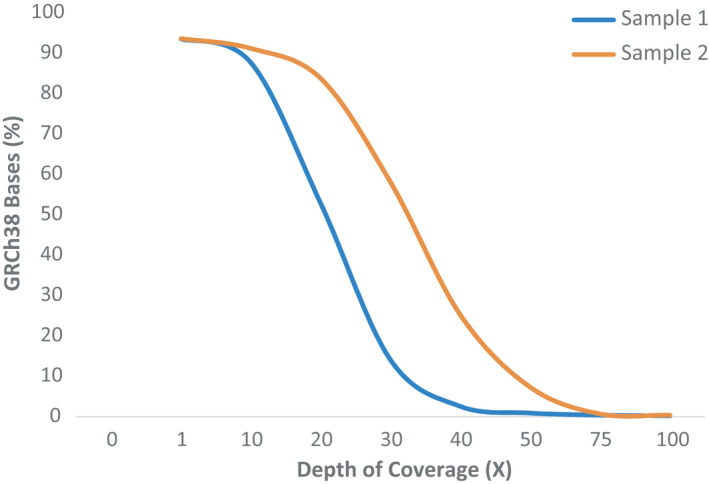
Coverage on the hg38 reference genome. The percentage of bases covered in the human GRCh38.p2 reference genome is shown at 1X, 10X, 20X, 30X, 50X, and 100X depth of coverage. Testing at 10X and 20X demonstrated sufficient coverage of the reference genome for accurate future downstream variant calls

Genomic coverage was assessed for each sample to determine the capture sensitivity. We analyzed genomic coverage at 1X, 10X, 20X, 30X, 40X, 50X, 75X, and 100X to examine what percentage of the genomic positions is adequately included at varying coverage thresholds (Figure 1). Accurate base sequencing calls are typically based on a minimum 10‐ to 20X‐fold depth of coverage (Lelieveld et al., 2015; Parla et al., 2011). Our results indicate that ~90% of the hg38 bases were covered with 10X or higher coverage in each sample. Competent base pair read coverage was demonstrated at 20X. Read coverage for sample 1 dropped to 53% but was acceptable with ~250 million deduplicated reads. Sample 2 retained confident coverage with 84% read coverage at 20X. Regions of the genome not covered by any reads were minimal with 6% of the bases not covered.

**FIGURE 2:**
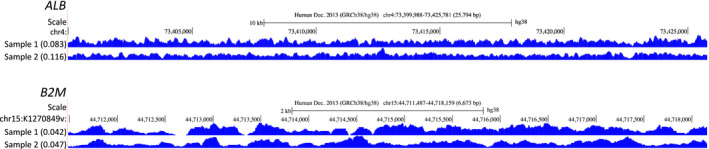
Parallel sample coverage of RPM normalized signal for the housekeeping genes, *ALB* and *B2M*

The Reads Per Million (RPM) normalized signal at various genes were reviewed to assess coverage consensus between the two samples (Figure 2). Coverage patterns for both samples paralleled each other. Figure 2 demonstrates raw base read coverage patterns along with normalized read scores for the housekeeping genes, albumin, (*ALB*) and beta‐2‐microglobulin, (*B2M*). Sample normalized read values were similar and consistent for each gene. *ALB* displayed high coverage with a normalized read score of ~0.01 RPM. In contrast to *ALB*, low base pair read coverage was observed for *B2M* and reflected in the similar, decreased normalized read scores (0.04 RPM).

**FIGURE 3:**
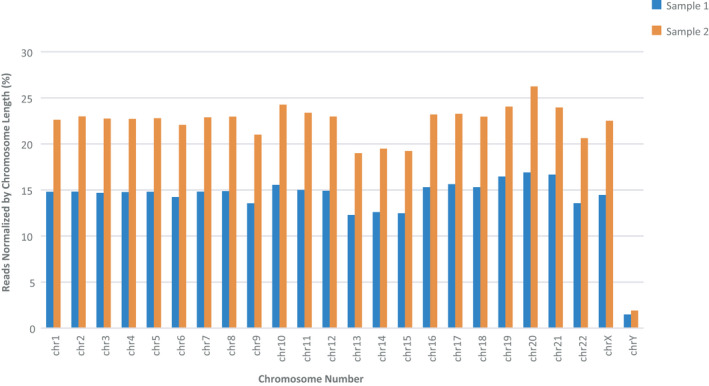
Distribution of coverage across each chromosome to determine the evidence of sequence bias. The percentage of aligned reads was normalized by chromosome length. For each sample, there is uniform coverage of each chromosome across the genome. Variant calls were performed using the GATK Pipeline (Broad)

We examined the distribution of coverage across each chromosome to determine the presence of sequencing bias by looking at the percentage of aligned reads normalized by chromosome length. For each sample, proportional to the number of aligned reads, there is uniform coverage of each chromosome across the genome (Figure 3). Of the five acrocentric chromosomes (Cingolani et al., 2012; Gehring et al., 2015; Hamaguchi et al., 2015; Plagnol et al., 2018; Zakrzewski et al., 2019), coverage was decreased for chromosomes 13–15. Annotated clone assembly problems for GRCh38.p2 have been described for the short arm regions of these chromosomes since they are heterochromatic and contain families of repeated sequences including ribosomal RNA gene arrays. The long arm is euchromatic and contains the protein‐coding genes of the chromosome (Dunham et al., 2004; Shepelev et al., 2015). Coverage for the Y‐sex chromosome is uniform, although chromosome coverage is substantially lower due to a sequencing gap in the reference genome. The majority (41 Mb) of the Y chromosome (63 Mb) is comprised of three blocks of highly reiterated satellites as well as other repeat sequences which complicates short read alignment (Kirsch et al., 2005).

Variants identified in repeat or blacklisted regions of the human genome were excluded from the analysis. Known variant calls (2,106,417 SNPs and indels) based on dbSNP build 151 (Sherry et al., 2001) accounted for 98.8% of the variants. The remaining 1.2% (25,277 SNPs and indels) were classified as “novel” with 0.002% identified as synonymous or non‐synonymous, missense mutations. Indels and non‐synonymous frameshift mutations accounted for the remaining novel variants.

**FIGURE 4:**
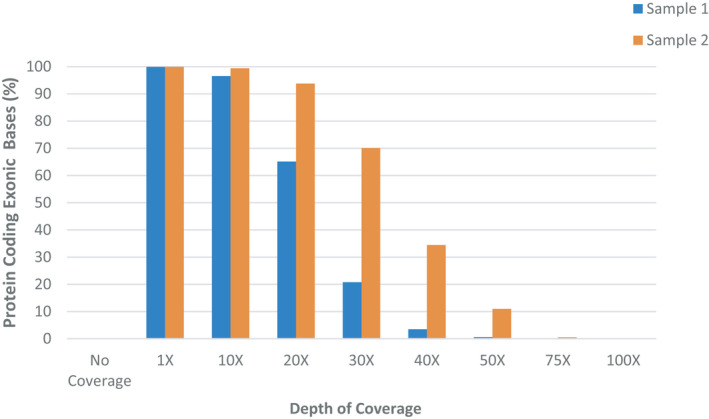
Coverage in protein coding exon bases from Agilent SureSelect® Human All Exon V7 probe design. The percentage of protein coding exon bases is shown at 1X, 10X, 20X, 30X, 50X, and 100X depth of coverage. Depth of coverage at 20X demonstrated sufficient coverage of the exonic bases for accurate, future downstream variant calls

WGS of amplified ccfDNA effectively accounted for all protein coding regions of the genome. We matched the coding exon sequences from this study to the well annotated Agilent SureSelect® Human All Exon V7 probe design. Exon probes target coding regions from RefSeq (99.3%), GENCODE v24 (99.6%), CCDS (99.6%), and UCSC known genes (99.6%) (https://www.agilent.com/cs/library/datasheets/public/5991‐9040EN%20SureSelect%20V7%20Datasheet.pdf ). Capture sensitivity was consistent across both samples (Figure 4). At a minimum coverage depth of 10X, >98% of the protein coding exon bases were sequenced for all samples. With increasing stringency at 20X, coverage decreased for both samples; however, sample 2 retained sufficient sensitivity (65%) to obtain valuable sequencing information. Protein coding exon bases not covered by any reads were negligible with less than 1% non‐covered bases.

**FIGURE 5:**
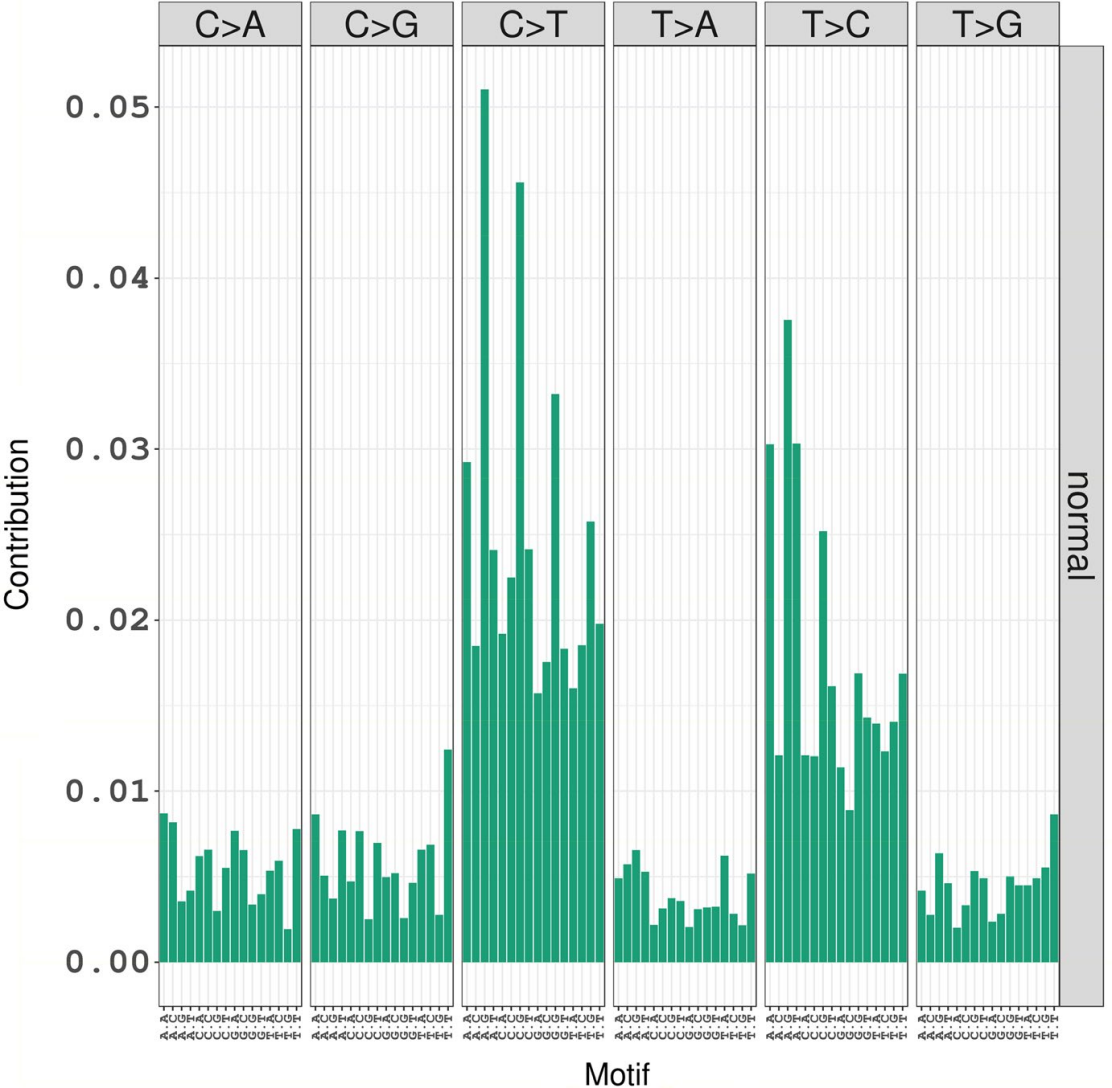
Mutational spectrum of the final variant set after excluding the low impact variants. Approximately two thirds of the variants were C>T and T>C mutations

We further characterized the final genomic variants where low impact variants were excluded with respect to the reference genome, GRCh38.p2, and generated a mutational frequency plot. A high percentage of C>T (39.9%) and T>C mutations (38.4%) was observed, Figure 5.

Spontaneous, 5‐methylcytosine deamination in resting cells creates point mutations from purine mismatching and leads to the frequently observed C>T transition mutations (Mustjoki & Young, 2021). We computed the frequency of each mutation in other known germline databases such as dbSNP (ftp://ftp.ncbi.nlm.nih.gov/snp/organisms/human_9606/VCF/GATK/All_20180418.vcf), HapMap and 1000 Genomes (https://console.cloud.google.com/storage/browser/genomics‐public‐data/resources/broad/hg38/v0/), and found that the percentage of C>T and T>C mutations in these resources matched with our study findings (Clarke et al., 2017; Gibbs et al., 2003).

In our final analysis, the final variant set was compared to two additional germline variant resource databases, gnomAD and 1000 Genomes Project (Clarke et al., 2017; Karczewski et al., 2020). When we compared our final variant set, concordance of 81.1% and 93.6% was found between our data set and that of the respective germline database. The high degree of overlap of shared variants between two germline genome resources demonstrates the potential capability of identifying variants from low input ccfDNA by WGS.

## 4 DISCUSSION

Clinical oncology research has recently been transformed by technological advancements to isolate, quantify, and sequence released cell‐free nucleic acids (DNA, RNA, and mtDNA) from a blood “liquid biopsy” (e.g., non‐small cell lung carcinoma) (Chen & Zhao, 2019; Malapelle et al., 2017; Plagnol et al., 2018). Since the half‐life of ccfDNA is between 16 min and 2.5 h, ccfDNA can mirror disease status in real time (Diehl et al., 2008). This carries the promise for future individualized diagnostic, prognostic, and therapeutic cancer applications, but technical challenges remain. Circulating cell‐free DNA concentrations are often inadequate in the absence of active disease or other factors that typically increase ccfDNA levels, and this can be further complicated by small volume plasma collections (≤ 4 ml) in clinical samples (Alborelli et al., 2019). As ccfDNA concentrations correlate with tumor size and stage, typically being lowest in patients with small tumors (Chen & Zhao, 2019), technical advances will be required for ccfDNA analysis to be useful in early stage cancer and other preclinical conditions. As a biomarker, ccfDNA concentrations have been disease informative, but downstream sequencing analysis could expand the utility of this molecule as a biomarker. In this study, we successfully isolated, amplified, and performed whole genome sequencing from low input amounts of plasma ccfDNA, thereby optimizing the methodology that may extend the range of ccfDNA analysis, and, thereby, its potential clinical and research utility.

Next‐generation sequencing in the form of targeted amplicon sequencing has been the primary approach to identify cancer‐related mutations from genomic DNA and ccfDNA samples. In the field of oncology, targeted amplicon sequencing has been extensively used for hereditary cancer screening, disease recurrence detection, or determining a therapeutic response (Marrugo‐Ramírez et al., 2018; Russano et al., 2020). Due to insufficient starting amounts of ccfDNA from vascular liquid biopsies, a limited number of studies have attempted whole genome or whole exome sequencing on these sample types. However, improvements with library preparation protocols for highly fragmented DNA samples have made WGS possible. We applied a library preparation method with reformulated repair and ligation reactions specific to plasma ccfDNA to successfully amplify and sequence the whole genome. Generation of blunt end fragments, followed by ligation and then extension, cleavage, and amplification were performed in a single tube to minimize the ccfDNA loss. We found base pair coverage was uniform across all chromosomes with enough reads for reliable variant calling, even though duplication rates were increased in one sample. Not unexpected, duplication rates do vary among samples as was found in this study. We observed a relatively high duplication rate (45%) for sample 1. Either low ccfDNA sample input (0.1 ng/μl in 10 μl), low library complexity, or the possibility of variance in ccfDNA fragment size with over representation of smaller fragments following PCR amplification were possible factors explaining the observed increased duplication rate. Samples were handled identically (duplication rate ≤30%) and fragment analysis indicated no differences in either sample quality until post‐amplification. By expanding the sequencing capabilities from gene targeting to include whole genome and whole exome sequencing, there is an acceleration of opportunities for disease discovery.

Few WGS studies have been conducted on clinical ccfDNA samples. Coverage of the genome at 10X and 20X was comparable to or better than other reports for WGS of ccfDNA (Wang et al., 2017). In a tumor ccfDNA matched study, Ma et al. (2017) validated that WGS of ccfDNA with low average coverage depth (~10X) was sufficient to detect variants from late stage lung and colon cancer samples with confidence (Ma et al., 2017). Our data at 20X coverage supports the reliability of sequenced calls from normal subjects with ~99% of the called variants present in dbSNP. The remaining variants absent in dbSNP are of unknown/uncertain significance (VUS, variants of unknown certainty). These variants could be attributed to clonal hematopoiesis and aging (Zink et al., 2017). Hematopoietic clone‐derived mutations including driver and passenger mutations are prevalent in ccfDNA of healthy individuals. VUS may be pathogenic or protective, an area that needs more investigation (Oulas et al., 2019). Those identified in this study will be followed up in subsequent work.

Many of the same single nucleotide variants are shared between germline and somatic mutation databases (Meyerson et al., 2020). Meyerson et al. (2020) found after strict filtering to exclude common germline polymorphisms and poor coverage or mapability sites, 336,987 common variants between the gnomAD germline database and the TCGA cancer genome database. They concluded that shared variants depict true biological occurrences of the same variant in the germline and somatic setting and arise primarily because DNA has some of the same basic chemical vulnerabilities in either setting. This helps to explain the mutation frequency pattern we observed from our variant data. A higher frequency of C>T and T>C transition mutations was present in the ccfDNA from the two normal liquid biopsy samples. This frequency has been reported frequently in the literature and resembles the COSMIC database clock‐like mutation signature, SBS5. Fiala and Diamandis (2020) reviewed mutations present in normal tissues and venous liquid biopsies in the context of developing specific ctDNA test for cancer, evaluating clonal hematopoiesis, cardiovascular pathology, and neural mosaicism in Alzheimer's disease (Fiala & Diamandis, 2020). In this review paper, work by Lodato et al. (2018), delineated three mutational signatures from single cell neurons isolated from the prefrontal cortex and hippocampus brain regions from healthy subjects and those diagnosed with early onset, hereditary neurodegenerative disorders, Cockayne syndrome, and Xeroderma pigmentosum (Lodato et al., 2018). The first signature, characterized by C>T and T>C mutations, increased with age, in a clock‐like fashion (Fiala & Diamandis, 2020; Lodato et al., 2018). The cancer genome database, COSMIC also reports this pattern as signature, SBS5 where approximately two thirds of the mutations are C>T and T>C. Even though COSMIC is a cancer genome database, the signature is described as clock‐like in that the number of mutations in most cancers and normal cells correlates with the age of the individual (https://cancer.sanger.ac.uk/signatures/sbs/sbs5/?genome=GRCh38). Our data from low input, plasma ccfDNA samples, directly correlate with germline resources and the well‐described COSMIC SBS5 signature for normal and cancer‐derived tissue.

Library preparation is a critical step in NGS sequencing and has a direct impact on the quality of sequencing results. Utilizing library preparation procedures specific to the amplification of highly fragmented plasma DNA, we successfully amplified ccfDNA and sequenced the whole human genome with uniform base pair distribution across each chromosome. Future work will continue to improve and refine the technique for clinical studies, and to translate the technique to exposure science, including both human and preclinical experimental animal model systems.

In summary, our results demonstrate that reliable data from WGS of low input clinical, ccfDNA samples can be used for biomarker and sequence variant discovery that can be applied to oncology, inflammation, transplantation, maternal/fetal disease, and other pathologies.

## CONFLICT OF INTERESTS

JFF, BE, BAM, KEG, MEC, JAM, GGS, MBF, and FWM are employees of the National Institute of Environmental Health Sciences (NIEHS) and National Institutes of Health (NIH), of the United States Government. The views expressed in this article are those of the authors and do not necessarily reflect the official policy or position of NIEHS, NIH, or the United States Government. DPP and RRS are Bioinformaticians at Sciome, LLC and performed WGS analysis and annotation under NIEHS contract HHSN273201700001C under NIEHS supervision. The NIEHS Epigenomics core facility (GGS) performed the genome sequencing. Sciome did not contribute to financial funding for publication of this work. The authors adhere to policies on public sharing of data and materials.

## AUTHORS' CONTRIBUTIONS

JFF, BE, BAM, and KEG made substantial contributions to the conception, design, and acquisition of data for the study. DPP and RSS conducted the bioinformatic analysis. MEC, JAM, and GGS provided NGS services through the NIEHS Epigenomics Core Laboratory. DPP, JFF, BE, BAM, and KEG contributed to the WGS data analysis and interpretation. MBF and FWM provided scientific study design input and review of the manuscript. All the co‐authors gave final approval for publication of the final version of the manuscript.
